# Plant growth promoting bacteria in the endo- and rhizosphere of halophyte *Cakile maritima* Scop.

**DOI:** 10.3389/fpls.2025.1672435

**Published:** 2025-11-12

**Authors:** Angela Guerrieri, Angela Racioppo, Antonio Bevilacqua, Giulia Conversa, Angelica Giancaspro, Barbara Speranza, Maria Rosaria Corbo

**Affiliations:** Department of the Science of Agriculture, Food, Natural Resources and Engineering, University of Foggia, Foggia, Italy

**Keywords:** plant growth promoting bacteria, rhizosphere, endosphere, halophytes, marginal areas, *Cakile maritima*

## Abstract

Beneficial bacteria adapted to hypersaline conditions represent a promising biotechnological tool for enhancing crop productivity in salt-affected agricultural systems, particularly as soil salinization constitutes an increasingly critical global challenge impacting extensive cultivated areas. Halotolerant Plant Growth Promoting Bacteria (PGPB) can stimulate plant growth by increasing soil carbon, nitrogen, and mineral availability and uptake. The present study aimed to isolate, characterize, and select potential halotolerant PGPB from *Cakile maritima* plants collected in two sites located in Margherita di Savoia, in Apulia. This coastal region harbors a unique and taxonomically diverse indigenous microbiota that remains largely unexplored, potentially serving as a valuable reservoir of beneficial microorganisms adapted to high salinity. Microbiological sampling was conducted in triplicate at three phenological stages (seeding, vegetative growth, and anthesis) of the plant’s life cycle in 2023. A total of 180 isolates (150 rhizobacteria and 30 endophytes) were recovered and characterized using morphological, biochemical, and molecular approaches. Halotolerant isolates exhibiting plant growth promoting traits, including phosphate and silicon solubilization, indole acetic acid and siderophore biosynthesis, ammonia production, and drought and salt tolerance, were selected and genotypically identified. The main result was the selection of three promising isolates (assigned to the genera *Pantoea* and *Bacillus*) warranting further validation under field conditions.

## 1 Introduction

The Mediterranean basin ranks among the regions most threatened by climate change globally (IUCN, 2008), with agricultural systems severely impacted by limited and highly variable rainfall distribution, increasing frequency of extreme weather events, and progressive soil and water salinization, which have resulted in prohibitive or economically unfeasible use of agricultural land (European Environment Agency, 2019). About 15% of the total land area of the world has been degraded through soil erosion and physicochemical deterioration, including soil salinization (marginal areas) (Wienhold, 2003; FAO, 2016).

Apulia, in Southeastern Italy, is characterized by extensive coastal zones and inland regions featuring naturally occurring saline environments, including salt marshes, coastal lagoons, and salt flats. The region’s distinctive geological and climatic conditions have generated diverse saline habitats that support specialized halophytic plant communities and their associated microbial ecosystems. Halophytes can grow at high levels of salinity, inhabiting naturally saline habitats or completing their life cycle at high salt concentrations (Flowers et al., 1986). These plants can tolerate salinity levels reaching 1 M NaCl and may exhibit enhanced growth in the presence of moderate salt concentrations (100–200 mM NaCl) (Flowers and Colmer, 2008; Shabala, 2013; Kumari et al., 2015). Due to this ability, halophytes thrive in a wide variety of saline habitats, ranging from coastal regions to marshes, as well as deserts, mudflats, salt flats, and steppes, where they play an important role in ecosystem protection due to their restoration ability (Flowers and Colmer, 2008). The environmental stress conditions, characteristic of highly saline soils, trigger specific biochemical pathways in halophytes, resulting in biosynthesis of secondary metabolites as adaptive responses (Qasim et al., 2017; Corrêa et al., 2020).

Microorganisms found in these habitats, in fact, possess specialized genetic and physiological attributes that allow them to survive and grow under harsh conditions, such as nutrient deficiency, reduced water availability, and excessive exposure to wind, rain, light, or high soil salinity (Agudelo et al., 2021; Brahim et al., 2021).

The microbiota associated with halophytic plants also includes halotolerant Plant Growth Promoting Bacteria (PGPB), which predominantly colonize the rhizosphere and endosphere (Mukhtar et al., 2018). Microorganisms capable of tolerating moderate to high salt concentrations could enhance plant growth and development by increasing the bioavailability and uptake of carbon, nitrogen, and essential minerals from the soil (Racioppo et al., 2023). Several studies have shown that PGPB isolated from the rhizosphere of halophytic plants can stimulate plant growth, increase crop yield, reduce pathogen infection, and alleviate both biotic and abiotic stresses without conferring pathogenicity (Accogli et al., 2023; Sharma et al., 2025). Due to their peculiar traits, such bacteria could represent an alternative strategy to enhance the salt tolerance of other crops and increase agricultural productivity.

Among halophytic plants, *Cakile maritima* Scop. (*Brassicaceae*), or sea rocket, grows in sandy coastal areas from the Arctic to the Mediterranean (Davy et al., 2006). This succulent annual plant reaches 10–30 cm in height, with ascending or prostrate stems. The leaves are fleshy and range from entire obovate to deeply pinnately lobed lamina. Inflorescences, in the form of dense racemes, terminate both the main stem and branches (Davy et al., 2006). Multiple studies have demonstrated that *C. maritima* enhances growth at moderate salinity levels (50–100 mM NaCl), exhibiting no adverse effects even at concentrations of up to 200 mM NaCl (Debez et al., 2004, 2018, 2012; Arbelet-Bonnin et al., 2019). In Italy, it grows wild in almost all regions, specifically, extensive sandy coasts. Near the village of Margherita di Savoia and the city of Barletta (BT province), it is present along the natural habitat represented by a sandy dunal cordon and in fields, called ‘*arenili*’, extending for 30 km alongside the sea (Conversa et al., 2020, 2024).

This plant can adapt to harsh environmental conditions such as lack of fresh water, wind and sandstorms, storm surges, dry soil, and high summer temperatures; it is also characterized by high content of iron, ascorbic acid, and iodine (Conversa et al., 2024).

Despite growing interest in plant-microbiome interactions, microbial community dynamics in halophyte plants growing in coastal environments remain poorly characterized. This knowledge is essential for understanding how these plants maintain beneficial microbial associations under fluctuating abiotic stress conditions, providing a first insight into their strategies for coping with salt stress, drought, and nutrient limitation—conditions that are increasingly relevant under climate change scenarios. Moreover, characterizing microbial dynamics in halophytes may reveal beneficial microorganisms with biotechnological potential for coastal agriculture, phytoremediation, and biosaline applications, thus bridging fundamental ecology and applied biotechnology in saline environments. Based on this rationale, the present study aimed to isolate, characterize, and select potential PGPB from *C. maritima* plants collected at two sites located in the coastal dunes of Margherita di Savoia (Apulia, Italy). Microbiological sampling was conducted in triplicate at three phenological stages (seedling, vegetative growth, and anthesis) throughout the 2023 growing season. This comprehensive temporal sampling approach was crucial in ensuring the isolation and identification of all microbial species that may colonize the plant during different growth phases, thereby providing a complete picture of the plant’s associated microbiome. In fact, plant-associated microbial communities undergo significant compositional and functional shifts throughout the host’s life cycle, driven by stage-specific physiological and biochemical changes (Chaparro et al., 2014). Each phenological stage is characterized by distinct root exudate profiles and nutrient availability, which selectively influence microbial colonization (Xu et al., 2018). For instance, the seedling stage represents the initial recruitment of microbes from the soil, and vegetative growth reflects the active root-microbe interactions that occur during biomass accumulation. At the same time, anthesis (flowering) involves shifts in resource allocation that alter the microbial community structure (Haichar et al., 2014). These changes are particularly pronounced in halophytic species, such as *C. maritima*, where salt tolerance strategies vary across developmental stages, potentially influencing the selection of stress-tolerant microbial partners (Vandenkoornhuyse et al., 2015). After isolation, all the obtained strains were characterized using morphological, biochemical, and genetic approaches.

## 2 Materials and methods

### 2.1 Sample collection and preparation

Microbiological sampling of *C. maritima* (sea rocket) was performed in triplicate at three phenological stages (seedling, vegetative growth, and anthesis) of the plant life cycle, during 2023. All samples were collected from two sites *viz.*, Margherita di Savoia (MdS) coastal dunes (S1: N 41.431624, E 16.008846; S5: N 41.427654, E 16.007945), in the Barletta-Andria-Trani area in northeastern Apulia (Italy). The soil at both sampled sites was predominantly characterized by sand (94%) with a low percentage of clay (6%), and a pH of 7.9; additional details are reported in **Supplementary Table S1**.

Each sample was collected using sterile gloves, forceps, spatulas, and scissors (Yamamoto et al., 2018). For each plant, two types of samples were collected: first, a 20x20 cm area at a depth of 10–20 cm was demarcated to collect the bulk soil (S); then, the plant was gently uprooted, including the entire root system. All collected material was stored in a portable refrigerator at 4°C, transported immediately to the laboratory, and refrigerated until further analysis. On the day of analysis, three samples were recovered: the first corresponded to the soil detached from the root by shaking, the second consisted of a 3 cm portion of the root (used for rhizosphere microorganism isolation), whereas the third one included the apical part of the plant and a small portion of the root (used for endophytic microorganism isolation).

### 2.2 Metagenomic profiling of soil

The soil samples (20 g) were subjected to shotgun metagenomic sequencing on the Illumina short-read NovaSeq platform (Aurogene s.r.l., Rome, Italy). For trimming, the fastP tool (v0.20.1) was used to preprocess the raw sequencing data (fastq) by filtering out adapter-contaminated and low-quality reads (below average quality 30, min. length 100 bp). The downstream bioinformatic analyses were performed on high-quality clean data by analyzing the filtered reads with Kraken2 (v2.1.2) for taxonomic classification, followed by Bracken (v2.9) for quantification of species abundance in DNA sequences. Bracken used the PlusPFP database (RefSeq archaea, bacteria, viral, plasmid, human1, UniVec_Core, protozoa, fungi & plant) (https://benlangmead.github.io/aws-indexes/k2) to estimate the number of reads originating from each species detected in the individual metagenomic samples. Taxonomic abundances obtained from Bracken outputs were visualized using the Krona tool (v2.8.1).

### 2.3 Endophytes isolation

Endophytes isolation was carried out following the method described by Christakis et al. (2021), with some modifications: this protocol, thanks to the surface sterilization procedures, ensures that the isolated strains originate exclusively from within the plant tissues. Specifically, plant material was repeatedly washed with sterile distilled water to remove soil and dust particles; then, for surface sterilization, plant roots and leaves were first shaken into sterile flasks containing 70% v/v ethanol for 60 s and then in flasks containing sodium hypochlorite solution 3% w/v (NaClO) for 10 min. The plant materials were then placed again in ethanol at 70% v/v for 60 s with shaking. To remove any remaining NaClO, plant materials were rinsed 10 times with sterile distilled water (dH_2_O). About 100 μL of the last rinse (for each analyzed sample) was plated on Nutrient Agar (NA) medium and monitored for microbial growth to evaluate surface sterilization efficiency. Only successfully sterilized root material was used further. Approximately 500 mg of leaves and roots per sampling were weighed and slashed into small parts for further processing using a sterile scalpel and further ground into a slurry with an autoclaved pestle and mortar. The slurry was transferred into sterile bottles, and 30 mL of autoclaved dH_2_O was added. The bottles were sealed and placed on a rotary shaker (150 rpm) at 25°C for 2 h. After shaking, 100 μl of the material in triplicate was inoculated on NA plates and incubated at 28°C. Colony-forming units were chosen from each plate based on their colour, texture, and morphology. Pure bacterial colonies were grown in Nutrient Broth (NB), and cell stocks were stored on NA slants at 4°C.

### 2.4 Isolation of rhizosphere microorganisms

For bacterial isolation from the rhizosphere at three distances (0 mm, 1–3 mm and 5–10 cm), 10 g of soil were diluted with 90 mL of a sterile saline solution (0.9% NaCl solution), homogenized in a Stomacher bag (Seward, London, England), and blended for 1 min in a Stomacher Lab Blender 400 (Seward). Then, serial dilutions were carried out and plated onto appropriate media, to select and count mesophilic bacteria (Nutrient agar; 30°C for 48 h), *Pseudomonas* spp. (Pseudomonas Agar Base added with Pseudomonas Selective Supplement; 25°C for 48–72 h), spore-forming bacteria (Plate Count Agar (PCA), after heat-treating the dilutions at 80°C for 10 min; the plates were incubated at 30°C for 24 h), Actinobacteria (Bacteriological peptone, 10 g/L; Beef extract, 5 g/L; NaCl, 5 g/L; Glycerol, 10 g/L; Agar, 20 g/L; 22°C for 7 days), and nitrogen-fixing bacteria (Glucose, 5 g/L; K_2_HPO_4_, 0.8 g/L; MgSO_4_, 0,2 g/L; FeSO_4_, 0,04 g/L; Na_2_MoO_4_, 0,005 g/L; CaCl_2_ anhydrous, 0,15 g/L; Agar, 15 g/L; 30°C for 5 days). All media and supplements were from Oxoid (Milan, Italy). From each plate, 5 to 10 colonies with different morphologies were randomly selected, isolated, purified, labeled with a numeric code, and stored at 4°C.

### 2.5 Characterization tests

All the isolates recovered from rhizo- and endosphere were morphologically and biochemically characterized through Gram staining, catalase, oxidase, urease test, microscopic observation, spore production, and motility (Olsen and Sommers, 1982). To assess the presence of plant growth promoting traits, the isolates were tested for their capacity to solubilize phosphate and silicon, produce indole acetic acid and siderophores, generate ammonia, promote nitrification, resist drought and high salinity.

#### 2.5.1 Plant growth-promoting bacteria traits

The ability of microbial isolates to produce ammonium was evaluated using the method described by Kumar et al. (2015), with some modifications. A 100 µL bacterial preculture was inoculated into test tubes containing Peptone Water medium (10.0 g peptone, 5.0 g NaCl, 1000 mL distilled water, pH 7.0). Tubes were incubated for 96 h at 30°C for mesophilic and spore-forming bacteria, 22°C for Actinobacteria, and 25°C for *Pseudomonas* spp. Ammonium production was determined by adding 0.5 mL of Nessler’s reagent to each tube. A pale-yellow color indicated low (+) ammonium production, while a yellow-brown or orange color indicated high (++) ammonium production.

Phosphate-solubilizing bacteria were assessed on Pikovsky medium (10 g/L d-glucose, 5 g/L Ca3(PO4)2, 0.5 g/L (NH4)2SO4, 0.2 g/L KCl, 20 g/L agar, 1000 mL distilled water, pH 7.0, Oxoid) following Dawwam et al. (2013). Plates were inoculated and incubated at 30°C for 48 hours. Phosphate solubilization was indicated by a clear zone around the inoculum; the halo diameter was measured in three directions, and the average calculated.

Siderophore production was evaluated using the method of Battini et al. (2016), with some modifications. Each microbial isolate was spot-inoculated (20 µL) on TSA (Tryptone Soy Agar, Oxoid) plates and incubated at 28°C for 48 hours. After incubation, 10 mL of CAS (Chromeazurol S) blue agar was overlaid on the plates and incubated at 28°C for 7 days. CAS blue agar was prepared according to Louden et al. (2011). Siderophore-producing strains were identified by a color change from blue to yellow or orange around the colonies. The halo was measured in millimeters (mm) from the edge of the colony to the outer edge of the colored zone. Strains were classified as follows: no production (0 mm), low production (< 2 mm), production (2 mm - 8 mm), moderate production (8 mm - 14 mm), and high production (> 15 mm).

The silicon solubilization test was performed using a specific medium containing 10 g/L of glucose, 2.5 g/L Mg_2_O_8_Si_3_, 20 g/L agar, and 1 L of distilled water. Bacterial strains were spot inoculated (20 µL) in the medium and incubated for 7 days at 28°C (Kang et al., 2017). After incubation, the presence of a transparent halo indicates the strain’s ability to solubilize silicon. The halo diameter was measured to estimate solubilization ability (Raturi et al., 2021).

Indole acetic acid (IAA) production was assessed using Yeast Extract Mannitol medium (YEM, 1 g/L Yeast extract, 10 g/L Mannitol, 0.5 g/L Dipotassium phosphate, 0.2 g/L Magnesium sulfate, 0.1 g/L Sodium chloride, 1 g/L Calcium carbonate, 0.1 g/L Tryptophan). Following the protocol of Dawwam et al. (2013) 250 µL of cell suspension was inoculated into 5 mL of YEM and incubated at 30°C. After 7 days, 1 mL of cell culture was transferred into a sterile Eppendorf tube and centrifuged at 10,000 rpm for 10 min; then, 500 µL of supernatant was mixed with 1 mL of Salkowski’s reagent and one drop of orthophosphoric acid (85%), and incubated at room temperature for 15 min. A pink coloration indicated the production of indole.

For nitrification, plates of Winogradsky medium (Chatterjee et al., 2015) were inoculated with fresh colonies and incubated at 30°C. After 48h, a drop of Griess’ reagent was added: the test is positive if, after a few seconds, the colonies change color to pink/purple.

#### 2.5.2 Salt and drought tolerance

Salt tolerance of all bacterial isolates was estimated based on their ability to grow at different concentrations of NaCl, ranging from 5% to 17.5% w/v in NA, incubated at 30°C. NA without salt served as the control. For each microbial isolate, plating was made on the agarized medium, and the plates were incubated at 30°C for 48 h. After incubation, colony development was checked to indicate the ability to grow at the appropriate salt concentration.

As suggested by Rashid et al. (2022) for the drought resistance tolerance test, the medium TSB (Trypticase Soya Broth, Oxoid) was modified by adding 5%, 10%, 15%, 20%, 25%, 30%, and 35% PEG6000 (polyethylene glycol). TSB without the addition of PEG6000 was used as a control. Inoculum (500 µL) was added to test tubes, incubated at 28°C for 48 hours under shaking at 120 rpm. After incubation, the absorbance was read at 600 nm with a UV-VIS spectrophotometer (Hach Lange, Milan, Italy). A medium with different percentages of PEG6000, without inoculation of microorganisms, was used as a control.

### 2.6 Identification of isolates

The most representative isolates were identified by sequencing the 16S rRNA. Briefly, total genomic DNA from the samples was extracted using the CTAB (Cetyltrimethylammonium Bromide) method, and the DNA concentration and purity were monitored on 1% agarose gels. According to the concentration, the DNA was diluted to 1 ng/μL using sterile water. 16S rRNA genes of distinct regions (16S V4/16S V3/16S V3-V4/16S V4-V5) were amplified using specific primers with the barcodes. Thermal cycling consisted of initial denaturation at 98°C for 1 min, followed by 30 cycles of denaturation at 98°C for 10 s, annealing at 50°C for 30 s, and elongation at 72°C for 30 s. Finally, 72°C for 5 min. The mixture of PCR products was purified with the Qiagen Gel Extraction Kit (Qiagen, Germany). Sequencing libraries were generated using the TruSeq^®^ DNA PCR-Free Sample Preparation Kit (Illumina, USA) following the manufacturer’s recommendations. The assembled sequences were compared with the reference database (Silva database (16S/18S), https://www.arb-silva.de/).

### 2.7 Statistical analyses

Metagenomic data (reads at family, genus, and species level) were used to evaluate α-(Dominance, Shannon, Simpson, Chao) and β-diversity indices and rarefaction curves through the software PAST, ver. 5.2.2 (https://www.nhm.uio.no/english/research/resources/past/).

The analyses were carried out in two independent batches. For each experiment, two technical replications were performed. The results of the qualitative analyses (ammonium production, phosphate solubilization, silicon solubilization, IAA production, siderophores) were converted into numeric codes (0, negative in all replicates of each isolate; 1, positive in all the replicates) and used as input values to run a principal component analysis; the Euclidean distance was used as the amalgamation method. This statistical analysis was performed using Statistica for Windows (StatSoft, Tulsa, OK, USA).

The results of the phenotypic test were also analyzed to find significant correlations through Spearman coefficients. Statistical analyses were done through PAST software.

## 3 Results

**Figure 1:**
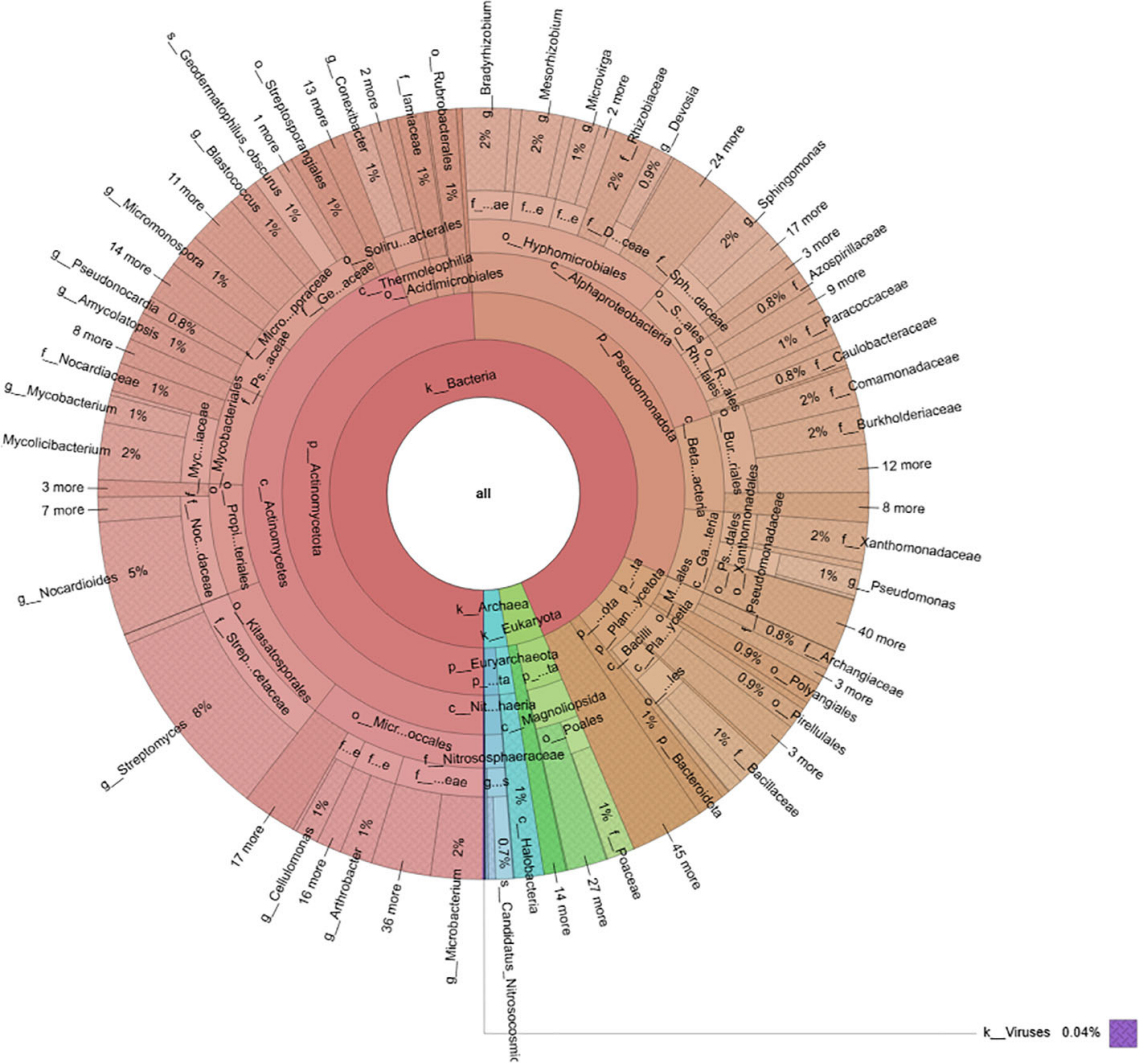
Circular phylogenetic tree illustrating the diversity and abundance of microbial taxa of the soil where *C. maritima* plants were collected.

Metagenomic analysis of the soil samples revealed a complex microbial community dominated by Bacteria (94%), followed by Eukaryota (4%) and Archaea (2%) (**Figure 1**). Bacteria belonged to two major phyla: Actinomycetota (52%) and Pseudomonadota (35%), which together accounted for 87% of the total bacterial diversity, whereas other bacterial phyla were less represented (13%).

**Figure 2:**
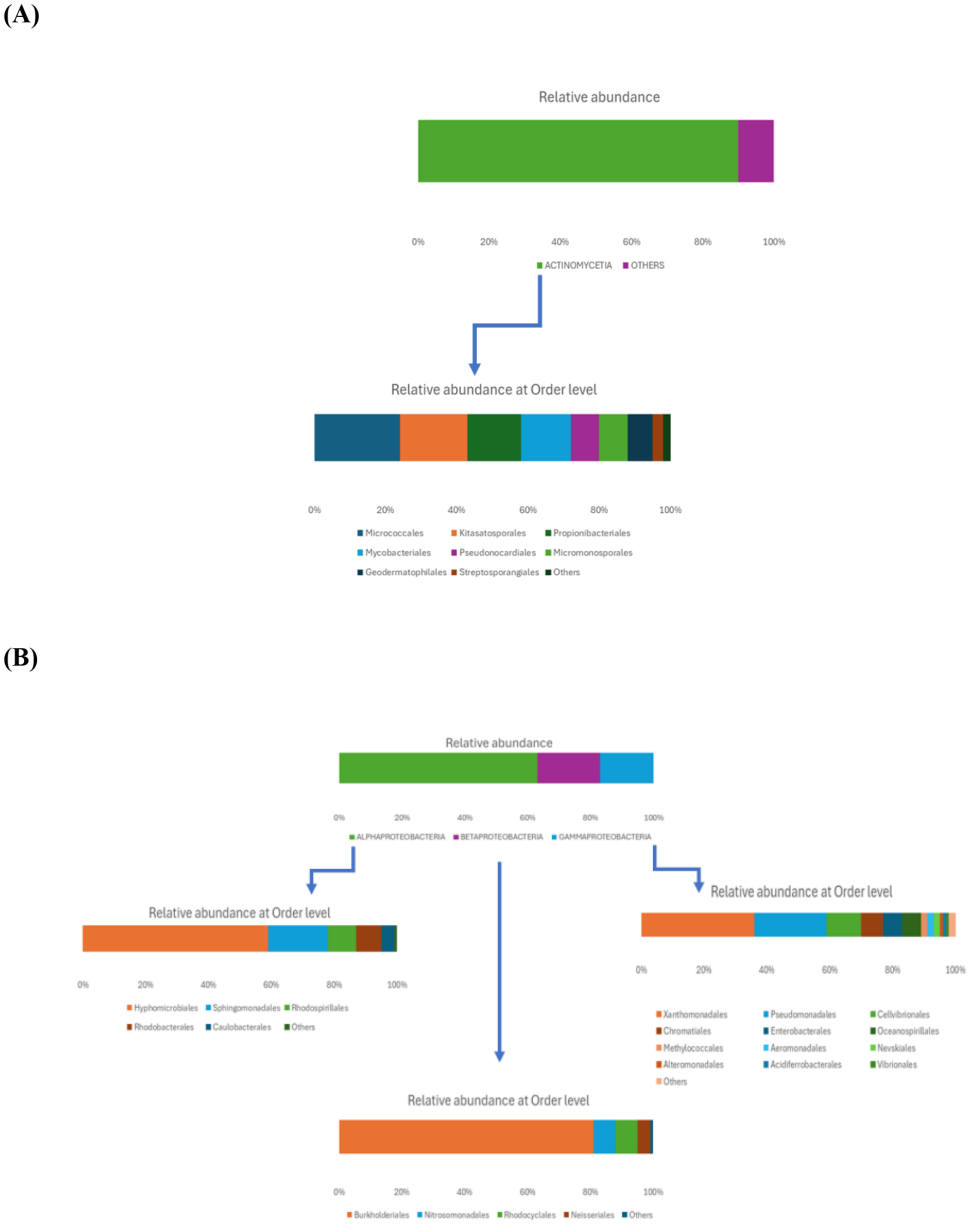
Relative abundance of rhizosphere soil bacterial communities at the class and order level within the Actinomycetota **(A)** and Pseudomonadota **(B)** phyla.

Within the Actinomycetota phylum, the predominant class was Actinomycetia (90%), which included several orders (**Figure 2A**): the most abundant one was Micrococcales (24%), followed by Kitasatosporales (19%), Propionibacteriales (15%), and Mycobacteriales (14%). Less representative orders included Pseudonocardiales and Micromonosporales (each at 8%), Geodermatophilales (7%), and Streptosporangiales (3%). The most prevalent families were Streptomycetaceae, Nocardioidaceae, Mycobacteriaceae, Microbacteriaceae, Streptosporangiaceae, and Micrococcaceae.

**Figure 3:**
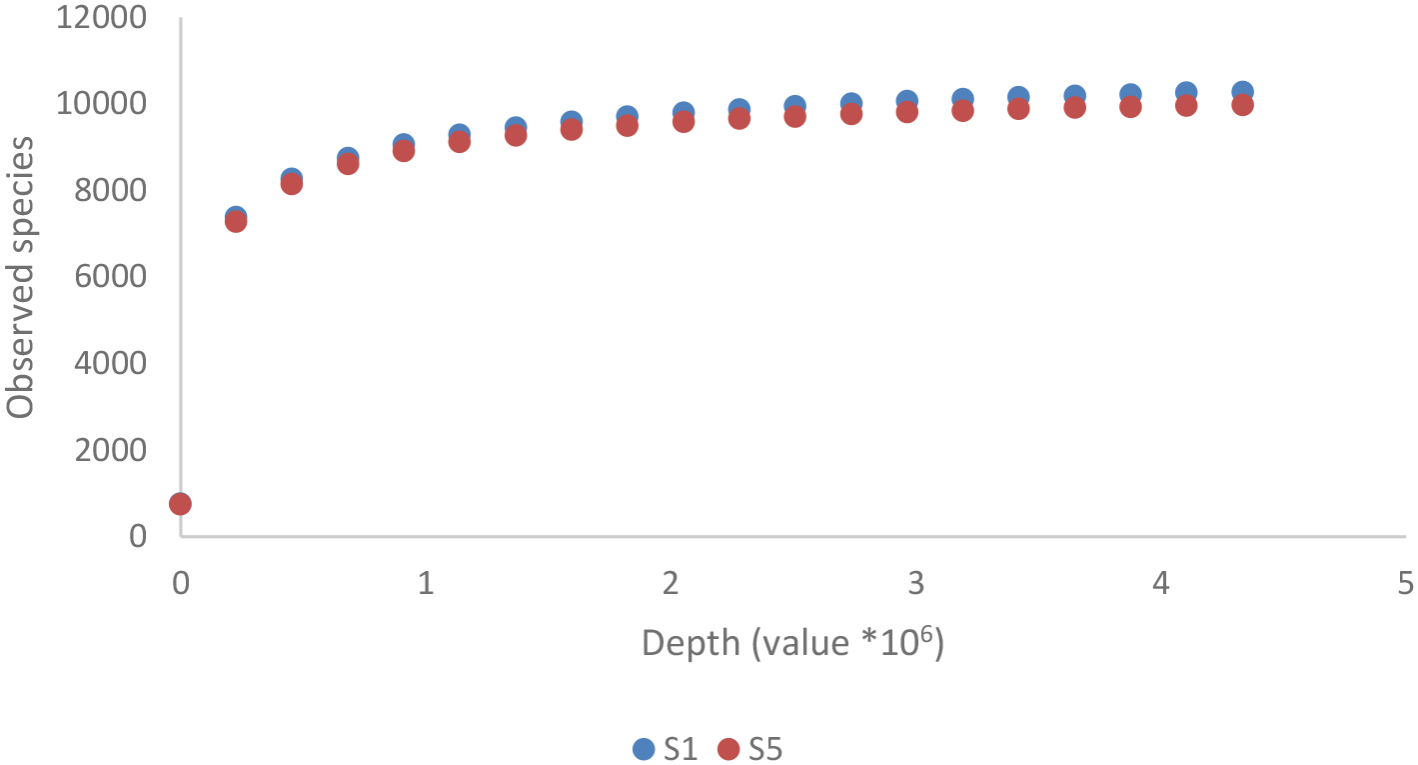
Rarefaction curve for the metagenomic analyses of the two soil samples. The sites (S1: N 41.431624, E 16.008846; S5: N 41.427654, E 16.007945) were both located in Margherita di Savoia coastal dunes (Barletta-Andria-Trani area in the northeastern Apulia, Italy).

**Figure 4:**
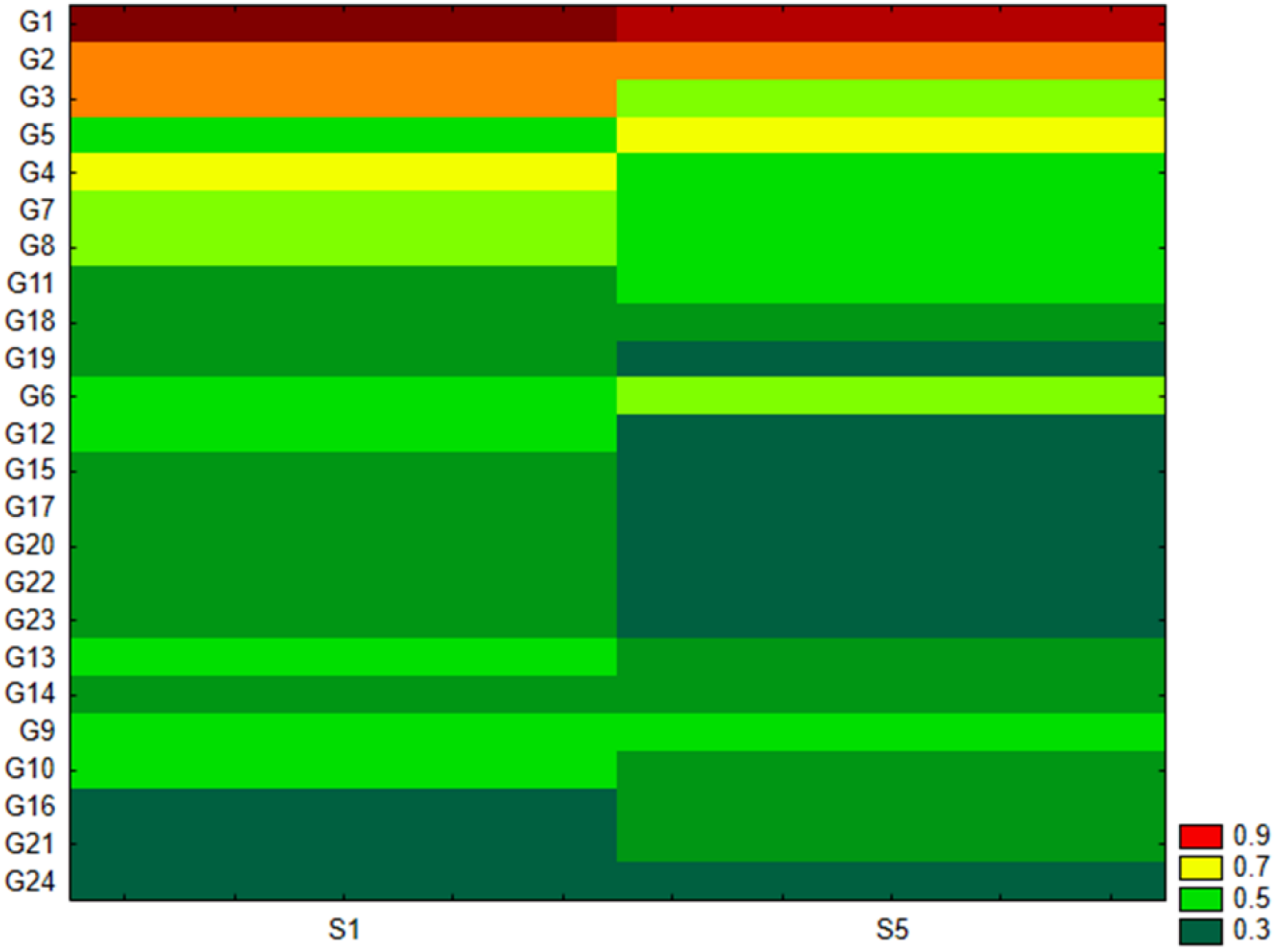
Heat map analysis for the top genera in the two soil samples. G1, *Geodermatophilus obscurus*; G2, Candidatus *Nitrosocosmicus oleophilus*; G3, *Blastococcus* sp. PRF04-17; G4, *Catellatospora* sp. IY07-71; G5, *Arthrobacter crystallopoietes*; G6, *Sorangium cellulosum*; G7, *Blastococcus saxobsidens*; G8, *Conexibacter woesei*; G9, *Capillimicrobium parvum*; G10, *Conexibacter* sp. SYSU D00693; G11, *Gemmatirosa kalamazoonensis*; G12, *Cellvibrio* sp. PSBB006; G13, *Modestobacter marinus*; G14, *Archangium gephyra*; G15, *Cellulosimicrobium cellulans*; G16, Candidatus *Nitrosocosmicus hydrocola*; G17, *Kocuria rosea*; G18, *Rubrobacter tropicus*; G19, *Baekduia alba*; G20, *Microvirga* sp. VF16; G21, *Nannocystis poenicansa*; G22, *Microvirga ossetica*; G23, *Iamia* sp. SCSIO 61187; G24, *Sandaracinus amylolyticus*.

As shown in **Figure 2B**, the Pseudomonadota phylum exhibited a tri-class distribution pattern. Alphaproteobacteria constituted the dominant class (63%), with Hyphomicrobiales as the most prevalent order (59%), followed by Sphingomonadales (19%), Rhodospirillales (9%), Rhodobacterales (8%), and Caulobacterales (4%). Betaproteobacteria represented 20% of the Pseudomonadota, predominantly composed of Burkholderiales (81%), followed by Nitrosomonadales (7%), Rhodocyclales (7%), and Neisseriales (4%). Finally, Gammaproteobacteria constituted 17% of the phylum Pseudomonadota, showing the highest diversity among the three classes with more than 13 orders: Xanthomonadales (36%), Pseudomonadales (23%), Cellvibrionales (11%), Chromatiales (7%), Enterobacterales (6%), Oceanospirillales (6%), Methylococcales (2%), Aeromonadales (2%), Nevskiales (2%), Alteromonadales (1%), Acidiferrobacterales (1%), and Vibrionales (0.8%). The Pseudomonadota phylum comprises several key families, with Pseudomonadaceae, Methylococcaceae, and Cellvibrionaceae being more prevalent, as well as Xanthomonadaceae, Sphingomonadaceae, Halomonadaceae, and Enterobacteriaceae. Metagenomic data were also used to calculate biodiversity indices and build rarefaction curves (**Figure 3**). The alpha diversity indices indicated that both soil samples had similar richness and evenness, with moderate values of Shannon (5.57 in S1 vs 5.65 in S5) and Simpson (0.014 in S1 and 0.015 in S5) diversity. The beta diversity analysis suggested possible differences in community composition (0.082 Bray-Curtis and 0.064 Jaccard). The rarefaction curves (**Figure 3**) showed a tendency toward a plateau, indicating that most of the species’diversity was captured, with a sufficient sequencing depth for analysis. The heatmap, constructed with the most abundant genera, shows the relative abundance of the top-genera (**Figure 4**). Briefly, the two samples showed a stable community with some prevailing genera, and a dynamic fraction with some differences. Namely, the most represented taxa were *Geodermatophilus* (G1) and Candidatus *Nitrosocosmicus oleophilus* (G2), suggesting their role as core members of the community. On the other hand, other genera, such as *Blastococcus* (G3) *Arthrobacter* (G5), *Catellatospora* (G4) and *Sorangium* (G6), showed different abundances between the two samples, reflecting differences in the structure of the microbial community.

To further characterize the cultivable fraction of this diverse microbial community, traditional plate count methods were employed using selective media. Mesophilic and spore-forming bacteria showed the highest cell load (ca. 7 log CFU/g), whereas actinobacteria, pseudomonads, and nitrogen-fixing bacteria were at lower levels (ca. 6 log CFU/g). The endophyte count amounted to 3 log CFU/g. From these microbial groups, a total of 180 bacterial isolates (150 rhizobacteria and 30 endophytes) were isolated and phenotypically characterized. As taxonomically expected, spore-forming bacteria were predominantly Gram-positive, whereas Pseudomonadaceae were predominantly Gram-negative. At least 50% of the putative mesophilic bacteria, nitrogen-fixing bacteria, and actinobacteria populations were found to be Gram-negative. Regarding hydrogen peroxide response, isolates from all microbial classes tested positive for catalase activity, a characteristic indicative of aerobic or aerotolerant metabolism. Pseudomonadaceae, which predominantly exhibit oxidative metabolism, were mainly oxidase-positive, as was a significant proportion of actinobacteria. Endophytic bacteria were mainly Gram-negative, oxidase-negative, and positive for catalase activity (data not shown).

**Figure 5:**
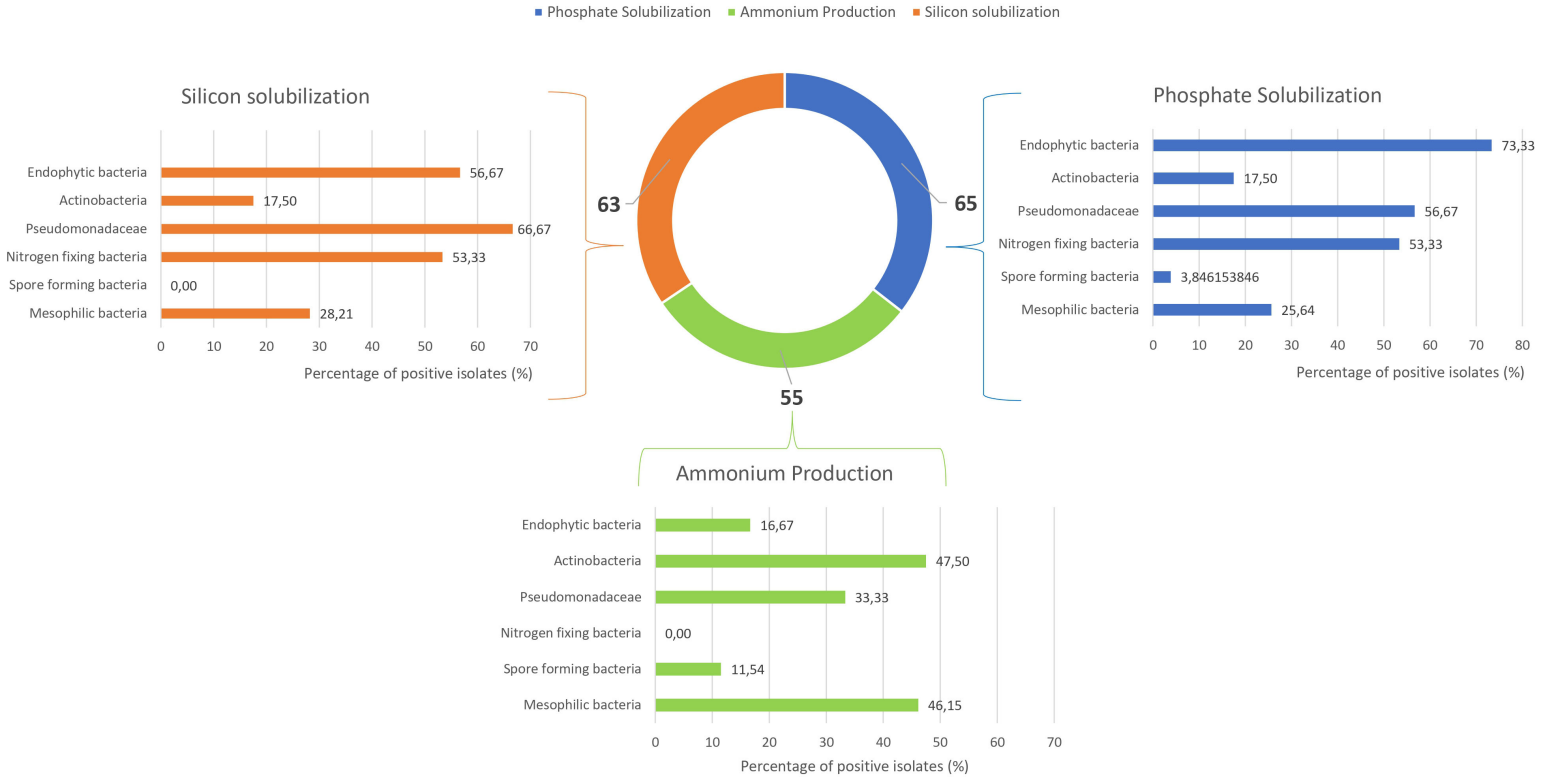
Bar and donut charts showing the percentage of positive strains for silicon solubilization, phosphate solubilization, and ammonium production.

The isolates were subsequently evaluated for key PGPB traits. **Figure 5** shows the results for phosphate and silicon solubilization assays, as well as ammonium production. Among the 180 microbial isolates tested, 65 demonstrated positive phosphate solubilization activity, with approximately 73% of endophytic bacteria positive for this assay. Pseudomonadaceae and nitrogen-fixing bacteria, with 57% and 53% of isolates, respectively, were also able to solubilize this compound. For silicon solubilization, 63 out of 180 strains exhibited positive results. Among these, Pseudomonadaceae (67%), endophytic bacteria (57%), and nitrogen-fixing bacteria (53%) showed the highest capacity. Ammonium production was observed at intermediate frequencies, with positive responses from 12% to 48% depending on the microbial groups.

**Figure 6:**
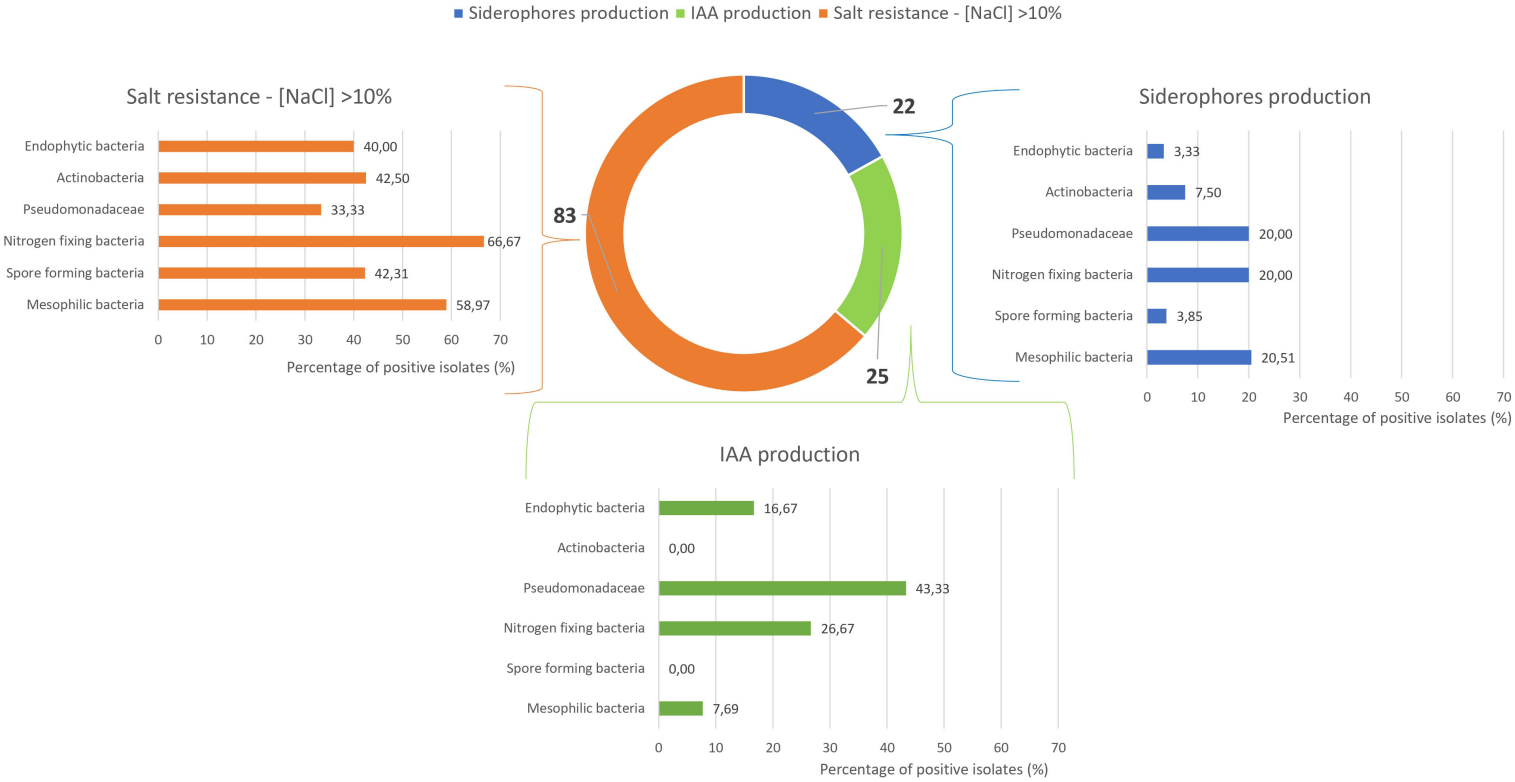
Strains resistant to NaCl >10% and positive for siderophores’ production, and Indole-3-Acetic Acid (IAA) production.

**Figure 6** provides an integrated overview of siderophore and indole-3-acetic acid (IAA) production, along with salt tolerance results. These, combined with the data in **Figure 5**, were used to identify potential PGPB isolates. Among the 180 microbial isolates examined, only 25 demonstrated IAA production capability, mainly presumptive Pseudomonadaceae (43%), while nitrogen-fixing bacteria, endophytic bacteria, and mesophilic bacteria showed limited IAA production ability (27%, 17%, and 8%, respectively). No isolates from actinobacteria or spore-forming bacteria groups demonstrated positive responses in this assay. Siderophore production was observed in only 22 out of 180 isolates, with all microbial groups showing moderate to low performance (positive isolates ranging from 3% to 20%).

A significant result was obtained regarding salt tolerance, as 83 strains demonstrated the ability to grow at salt concentrations exceeding 10%. Among these, nitrogen-fixing bacteria exhibited the highest percentage of positive isolates (67%), followed by mesophilic bacteria (59%). Spore-forming bacteria, actinobacteria, and endophytic bacteria demonstrated salt tolerance in approximately 40% of isolates, while presumptive Pseudomonadaceae exhibited salt tolerance in 33% of tested strains. As better highlighted in **Supplementary Figure 1**, 83 strains demonstrated the capacity to grow at salt concentrations exceeding 10%. Among these, 35 strains showed the ability to thrive at salt concentrations above 15%, while 20 strains displayed exceptional resilience, tolerating salt concentrations as high as 17.5%.

Some of the isolated strains were able to develop even under drought stress conditions, recording growth indices of up to 60% compared to optimal growth conditions; the most promising results were obtained for the endophytes 11CE, 14CE, and 15CE and the rhizobacteria 19C, 42C, 134C, and 178C (data not shown).

Selected phenotypic assays were further analyzed to assess possible correlations among traits. Nitrification was excluded, as it was mostly negative, while for salt tolerance, only growth in the presence of 10% was retained, as at higher concentrations, the number of positive strains was very low, and which could have affected the strength and the significance of correlations. The results are in **Supplementary Figure 2**. Statistical analysis revealed a significant negative correlation between salt and phosphate: for 49% of isolates the two traits were mutually exclusive, as isolates capable of solubilizing phosphate were unable to grow in the presence of salt. Conversely, positive correlations were observed, with coefficients ranging from 0.43 to 0.49, between silicon and phosphate solubilization, silicon and IAA production, and IAA and phosphate.

A correlation matrix is an important tool to assess possible links among different variables, but it cannot be used to select promising isolates for a process optimization. This objective could be achieved through other multivariate analyses, such as Principal Component Analysis.

**Figure 7:**
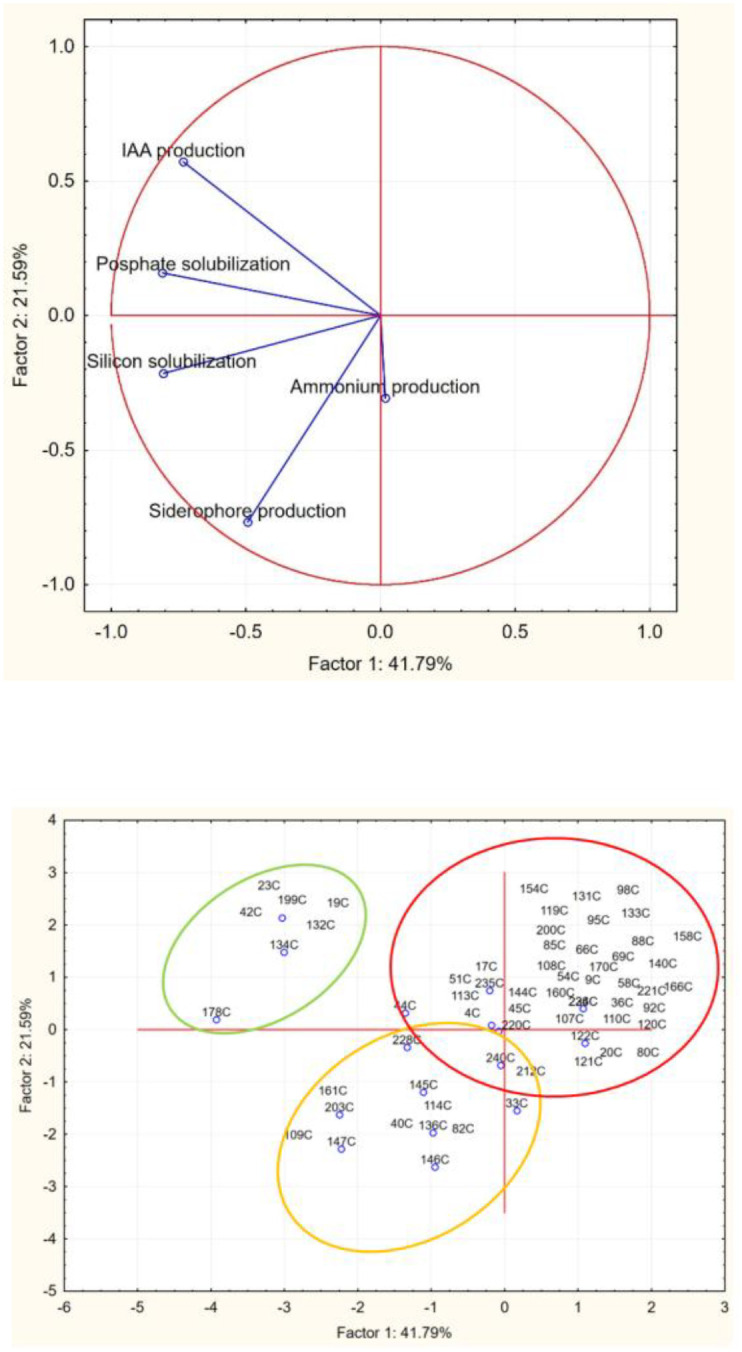
Principal component analysis run on halotolerant bacteria (growth at NaCl>10%).

Therefore, to select the most promising microorganisms with PGPB traits, results from qualitative analyses, including assessments of phosphate and silicon solubilization, IAA production, siderophore production, and ammonium production, were converted into numerical codes and used as input values for principal component analysis (**Figure 7**).

As demonstrated in **Figure 7**, data analysis enabled the classification of strains into three distinct groups, with the best performers being those in the upper-left cluster, which includes the seven most promising PGPB strains. Among these strains, three isolates were selected based on their comprehensive PGPB profiles: isolates 42C and 178C, identified as *Pantoea agglomerans* and *Pantoea* sp., respectively. demonstrated all tested traits except ammonium and siderophore production, respectively, whereas isolate 134C (identified as *Bacillus* sp.) exhibited all PGPB characteristics except siderophore production (**Table 1**).

**Table 1:** Identification and PGPB (Plant Growth Promoting Bacteria) characteristics of the isolates selected.

Isolate	Identification	GenBank accession number	Ammonium production	Phosphate solubilization	Salt resistance (>10%)	Silicon solubilization	IAA production	Drought resistance	Siderophores production
42C	*Pantoea agglomerans*	PX273518	–	+	+	+	+	+	–
134C	*Bacillus* sp.	PX258216	+	+	+	+	+	+	–
178C	*Pantoea* sp.	PX273912	–	+	+	+	+	+	+

## 4 Discussion

The rhizosphere is a critical ecological zone that facilitates nutrient exchange between plants and their surrounding environment (Yin et al., 2021). As the most abundant microbial group in soil - representing 94% of the microbial community, including in this study - bacteria play a central role in maintaining the functionality of this complex ecosystem (Zhou et al., 2023).

In this study, the microbial community structure appeared highly heterogeneous. The dominance of Actinomycetota (particularly orders such as Micrococcales, Kitasatosporales, and Streptosporangiales) and Pseudomonadota indicates a significant metabolic versatility within the soil microbiome, including nitrogen fixation, mineral solubilization, and other essential functions (Ma et al., 2021). At the phylogenetic level, Actinobacteria and Gammaproteobacteria were highly abundant, confirming findings by Marasco et al. (2016), who isolated PGPB from the rhizosphere of the halophyte *Salicornia strobilacea* in Tunisian coastal areas. These authors selected two rhizobacteria (belonging to the *Pseudomonas* and *Bacillus* genera) able to (i) resist an array of abiotic stresses typical of extreme environments, (ii) produce plant hormones, and (iii) stably colonize plant root. Other studies have reported similar bacterial communities and isolated strains capable of improving plant growth under saline conditions (Sadeghi et al., 2012; Bodenhausen et al., 2013; Mukhtar et al., 2017; Etesami and Beattie, 2018; Yamamoto et al., 2018; Kearl et al., 2019; Yin et al., 2021).

The high taxonomic diversity within dominant phyla, together with metabolically specialized orders, reflects a well-structured soil microbial community potentially rich in PGPB traits. This justified the isolation and characterization of bacterial strains. Among the 180 isolates, the predominance of Gram-positive spore-forming bacteria and Gram-negative Pseudomonadaceae agrees with previous findings in saline environments, where these bacterial groups are known to thrive due to their robust stress tolerance mechanisms (Etesami and Beattie, 2018). The result found for phosphate solubilization (65 out of 180) is particularly significant, as phosphorus availability is often limited in saline soils due to precipitation with calcium and magnesium ions. Notably, endophytic bacteria capable of solubilizing this compound suggest that these internal plant-associated microorganisms may play a crucial role in phosphorus nutrition under saline conditions. This result agrees with recent studies highlighting interesting PGPB activities of endophytes compared to rhizosphere bacteria (Santoyo et al., 2021). The strong efficiency of presumptive Pseudomonadaceae (57% positive) in phosphate solubilization is already well-documented in various environments (Santoyo et al., 2021). This trait, combined with their salt tolerance, makes them particularly valuable for agricultural applications in saline soils.

Silicon solubilization, observed in 35% of isolates (63 out of 180), is another important finding. Silicon enhances plant resistance to abiotic stresses such as salinity by strengthening cell walls and improving water use efficiency, making it a crucial factor in plant stress tolerance under saline conditions (Zhu and Gong, 2014).

The limited number of isolates capable of IAA production (25 out of 180, 14%) is not surprising due to the specialized nature of this trait; IAA production is crucial for root development and stress adaptation, making these isolates particularly valuable for enhancing plant establishment in challenging conditions (Spaepen et al., 2007).

The most significant finding is the high salt tolerance exhibited by 46% of isolates (83 out of 180), able to grow at concentrations exceeding 10%. The exceptional performance of nitrogen-fixing bacteria (67% positive) is particularly noteworthy, as these organisms can simultaneously provide nitrogen nutrition and salt stress mitigation. The fact that 20 strains tolerated salt concentrations as high as 17.5% indicates the presence of highly specialized halotolerant mechanisms that could be exploited for extreme saline conditions.

The above-mentioned results demonstrate that halophytic plants like *C. maritima* may serve as valuable reservoirs of halotolerant PGPB with multiple beneficial traits. However, the selection of promising microorganisms represents a multifaceted challenge that requires careful consideration of the inherent trade-offs between desirable and less favorable traits. It is essential to recognize that microorganism selection constitutes a risk-benefit analysis, acknowledging that no “super-organism” exists with all characteristics at their optimal levels. Instead, the selection process involves strategic compromises among various traits to identify the most suitable candidates for specific applications. The complexity of microorganism selection stems from the need to manage extensive datasets encompassing numerous strains (180 strains), each characterized by multiple variables (8 different tests were performed) that may be qualitative or quantitative, binary or multidimensional, etc. This multidimensional nature of the data presents significant analytical challenges that require sophisticated statistical approaches.

Two primary challenges in this selection are: 1) reducing data complexity while preserving critical information and 2) defining inclusion and exclusion criteria to refine the candidate pool without excluding valuable strains or allowing excessive ambiguity.

A range of data clustering and classification techniques can be employed for this purpose, including cluster analysis, principal component analysis (PCA), k-means clustering, and multiple correspondence analysis. Each approach offers distinct advantages and limitations depending on the specific research context and data characteristics.

Principal component analysis emerges as the most appropriate technique for this research based on three key advantages: (1) it effectively reduces the dimensionality of large variable sets while minimizing information loss; (2) it facilitates the division of samples into homogeneous groups through effective clustering; and (3) it offers insights into the main variables influencing the clustering process, allowing for the identification of the most significant traits involved in the selection. The initial step in implementing this approach involves clearly defining the research objective: in this case, the selection of promising PGPB with optimal trait combinations for biotechnological applications. Focusing on salt resistance, phosphate and silicon solubilization, IAA production, siderophore production, and ammonium production, the principal component analysis successfully identified distinct bacterial groups based on their PGPB trait profiles, with the selection of three promising strains (42C, 134C, and 178C) representing isolates with complementary capabilities. These strains belong to the genera *Pantoea* and *Bacillus*, both well-known for PGPB activities and environmental adaptability (Gouda et al., 2018; Santoyo et al., 2021). *Pantoea* species exhibit versatile PGPB traits, including phosphate solubilization, IAA production, and stress tolerance (Walterson and Stavrinides, 2015). *Bacillus* species, particularly spore-forming strains, offer advantages in terms of environmental persistence and bioformulation stability, making them ideal candidates for commercial applications (Meena et al., 2017). By combining classical microbiological approaches with advanced metagenomic sequencing, this study provided a more complete overview of the structure and function of soil bacterial communities of *C. maritima*. The use of high-throughput technologies combined with bioinformatic pipelines for taxonomic classification enhances the understanding of complex samples and paves the way to better exploitation of plant-microbe interactions for several goals, such as adaptation to climate change, reduction of fertilizer input, and the replacement of chemical pesticides with less hazardous alternatives.

## 5 Conclusion

The obtained results demonstrate that halophytic plants like *C. maritima* may serve as valuable reservoirs of halotolerant PGPB with multiple beneficial traits. The combination of salt tolerance with multiple PGPB traits in the selected isolates suggests their potential application in salt-affected agricultural lands. These bacteria could be used as biofertilizers to improve crop productivity while reducing the need for chemical fertilizers and soil amendments.

Nevertheless, although this study provides a comprehensive screening of PGPB traits, the transition from laboratory-based characterization to field application requires validation under realistic agricultural conditions. Future research should focus on field validation of the selected strains under various crop systems and salinity levels, and on interaction studies to evaluate compatibility with existing soil microbiomes and potential synergistic effects. Moreover, a better understanding of the molecular basis of salt tolerance and PGPB activities should be useful.

## Data Availability

The raw data supporting the conclusions of this article will be made available by the authors, without undue reservation.
