# Supplementary material for: Plant growth promoting bacteria in the endo- and rhizosphere of halophyte Cakile maritima Scop
Source: Front Plant Sci. 2025 Nov 12;16:1672435. doi: 10.3389/fpls.2025.1672435 (PMC12648727; doi:10.3389/fpls.2025.1672435)
Supplement: Supplementary file 1 [file DataSheet1.pdf]

Supplementary Material

Supplementary Figure 1. Salt-tolerant strains at increasing concentrations of NaCl (10%, 15%, and 17.5%).

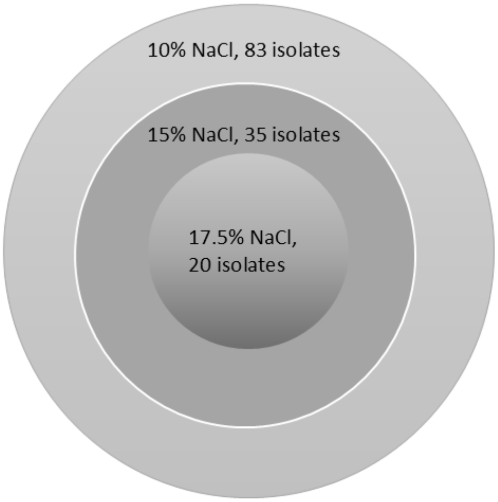

Supplementary Figure 2. Correlation matrix among the phenotypic tests. Amm, NH_4^+ production; Phosp, phosphate solubilization; NaCl, growth in presence of 10% NaCl; Si, silicon solubilization; IAA, indole acetic acid production; Sid, siderophore production. The correlation coefficients range from –1 to +1 and are color-coded from blue (negative correlation, -1) to red (positive correlation, +1). Grey cells highlight significant correlations.

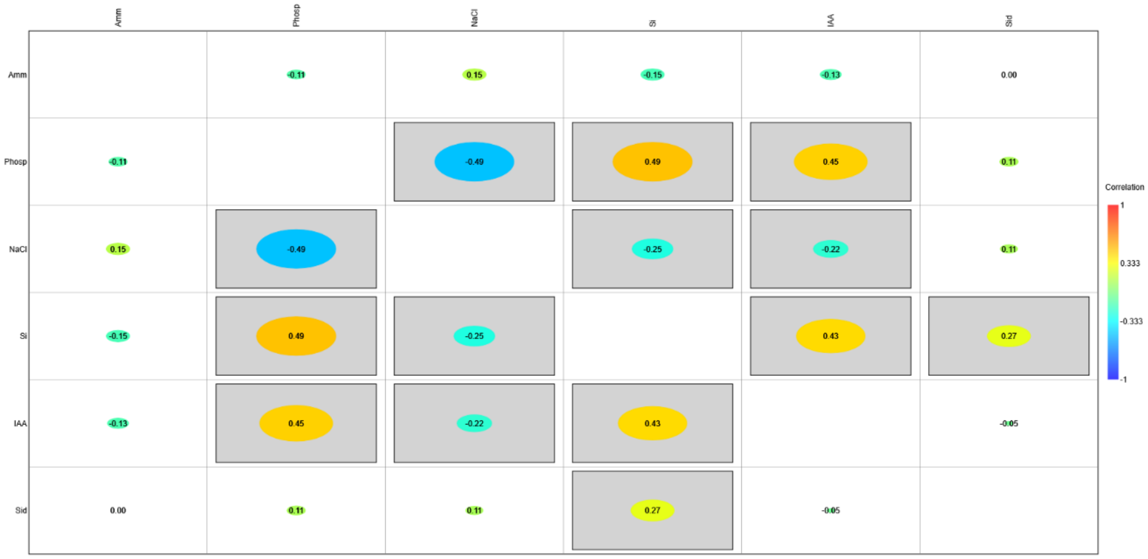

Supplementary Table 1. Physico-chemical characteristics of the soil where *Cakile maritima* plants were collected (Margherita di Savoia, BAT, northern Puglia).

Determination	Result	Determination	Result
**Bulk density (kg/ m^3)**	1470	**Available magnesium (Mg) (mg/kg)**	43
**Silt (%)**	0	**Available sulfur (S) (mg/kg)**	21.2
**Sand (%)**	93.8	**Available sodium (Na) (mg/kg)**	48
**Clay (%)**	6.2	**Assimilable iron (Fe) (µg/kg)**	< 2010
**pH**	7.9	**Available silicon (Si) (µg/kg)**	24030
**Electrical conductivity (dS/m)**	9.5	**Assimilable manganese (Mn) (µg/kg)**	470
**Organic matter (g/100g)**	0.5	**Assimilable zinc (Zn) (µg/kg)**	< 100
**Total nitrogen (g/kg)**	0.48	**Assimilable copper (Cu) (µg/kg)**	33
**Total potassium (mmol/ kg)**	0.9	**Soluble boron (B) (µg/kg)**	212
**Exchangeable potassium (K_2O) (mg/kg)**	50.6	**Available cobalt (Co) (µg/kg)**	< 2.6
**Total phosphorus (P_2O_5) (mg/kg)**	1090	**Available molybdenum (Mo) (µg/kg)**	3
**Available phosphorus (P_2O_5) (mg/kg)**	8.5	**Cation exchange capacity (mmol/kg)**	24
**Available calcium (Ca) (mg/kg)**	79	**Microbial biomass (mg C/kg)**	32
